# The Stop Transmission of Polio Data Management (STOP DM) assignment and its role in polio eradication and immunization data improvement in Africa

**DOI:** 10.11604/pamj.supp.2017.27.3.11524

**Published:** 2017-06-22

**Authors:** Amalia Benke, Alford Joseph Williams, Adam MacNeil

**Affiliations:** 1 The Centers for Disease Control and Prevention, Atlanta, GA, USA

**Keywords:** Immunization, surveillance, data, polio, workforce

## Abstract

The availability and use of high quality immunization and surveillance data are crucial for monitoring all components of the Global Polio Eradication Program (GPEI). The Stop Transmission of Polio (STOP) program was initiated in 1999 to train and mobilize human resources to provide technical support to polio endemic and at-risk countries and in 2002 the STOP data management (STOP DM) deployment was created to provide capacity development in the area of data management for immunization and surveillance data for these countries. Since 2002, Africa has received the majority of support from the STOP DM program, with almost 80% of assignments being placed in African countries. The STOP DM program has played a valuable role in improving the quality and use of data for the GPEI and has increasingly supported other immunization program data needs. In this report we provide an overview of the history, current status, and future of the STOP DM program, with a specific focus on the African continent.

## Brief

### Introduction

Since the inception of the Global Polio Eradication Initiative (GPEI) in 1988, the number of annual cases of paralytic poliomyelitis has decreased from 350,000 annually to less than 40 in 2016 and only 3 countries currently have endemic transmission of wild polio virus (WPV) [1-3]. Similarly, during this time period, WPV went from being endemic across virtually the entire continent of Africa to endemic in only one country (Nigeria) [4]. Well-functioning immunization information systems and high quality integrated epidemiological and laboratory surveillance are crucial components of the GPEI and necessary for ensuring optimal program performance at all levels of the public health system, from health facilities, to national programs, to global partners. Due to the complexity of the GPEI, numerous sources of polio data are used, including routine vaccination coverage, case-based surveillance, laboratory testing, immunization campaign monitoring and post-campaign coverage, vaccine stocks, and financing. Despite the need for these data to optimize immunization program performance, major challenges in the quality and use of immunization and surveillance data, including polio data, have been well recognized for many years [5, 6].

### The STOP and STOP DM Programs

The Stop Transmission of Polio (STOP) program was initiated in 1999 by the Centers for Disease Control and Prevention (CDC), in collaboration with the World Health Organization (WHO) to train and mobilize human resources to provide technical support to polio endemic and at-risk countries [7]. Since its creation, the STOP program has evolved into a large initiative, in which participants are recruited globally and deployed as WHO or UNICEF volunteers to countries for 5-month deployments (prior to 2012, these were 3-month deployments), with volunteers being allowed to deploy for up to four consecutive assignments in the same country. The majority of STOP volunteers operate at lower levels (e.g., district) of their assigned country. From 1999 through September, 2016 48 STOP teams, with 1829 total participants have been deployed on 3395 total assignments. During this time period, 1339 participants (on 2562 assignments) deployed to 44 countries (or WHO regional offices) in Africa.

Due to well recognized issues with the availability, quality, and technical skills to manage and use polio immunization and acute flaccid paralysis (AFP) surveillance data, the STOP data management (DM) assignment was created in 2002 as a mechanism to provide capacity development in the area of data management. Through the addition of the STOP DM assignment to the STOP program, STOP DM participants are recruited through the same core process as the overall STOP program. The assigned terms-of-reference are specific to a country’s data needs; assignees typically are placed at the national level, either in the Ministry of Health or country WHO office. Since 2002, a total of 172 STOP DM participants (316 assignments) have been deployed, with 79% (n=250)) assignments to country or WHO regional offices in Africa. STOP DM participants have been placed in a total of 38 African countries and the overall number of deployments to Africa has increased substantially from 6 in 2002 to 46 in 2016. STOP DM participants commonly participated in multiple consecutive assignments in their assignment location, with an average of 1.95 assignments per participant.

### Recruitment, training, and activities of STOP DM

Recruitment of qualified candidates for the STOP DM position is a competitive process and only a small proportion of applicants end up deploying to the field. To be eligible to apply, STOP DM applicants are required to have extensive experience working within and supporting the health information system, and at least 5 years of experience as an immunization or surveillance data manager. Candidates must also have a Master’s degree, or more than 12 years of experience working in the field of public health.

Accepted STOP DMs are required to attend three weeks of training before they are deployed on their first assignment. The first two weeks of training sessions are focused on polio eradication, strengthening routine immunization and vaccine-preventable disease (VPD) surveillance, as well as supporting monitoring and implementation of supplemental immunization activities. The STOP Data Management training facilitated during the third week emphasizes a systems-focused approach to improving immunization and VPD surveillance data quality to develop system and human resource capacity for data utilization and strengthening information systems. The participatory training leverages the knowledge, skills and experiences of STOP DMs through facilitation of practical sessions focused on immunization and VPD surveillance data management processes, data flow, data quality, root cause analysis, giving and receiving constructive feedback, development of recommendations and effective communication and visualization of data.

**Figure 1:**
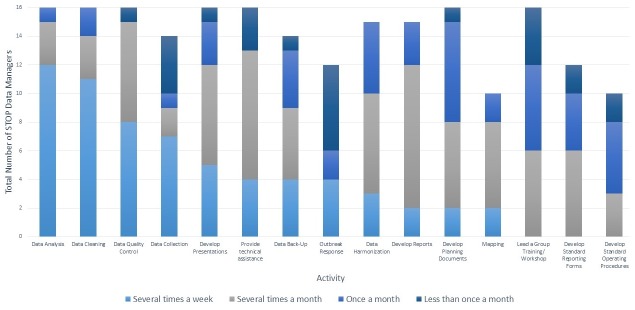
The proportion of STOP deployment number 44 data managers reporting to support data management activities by frequency

**Figure 2:**
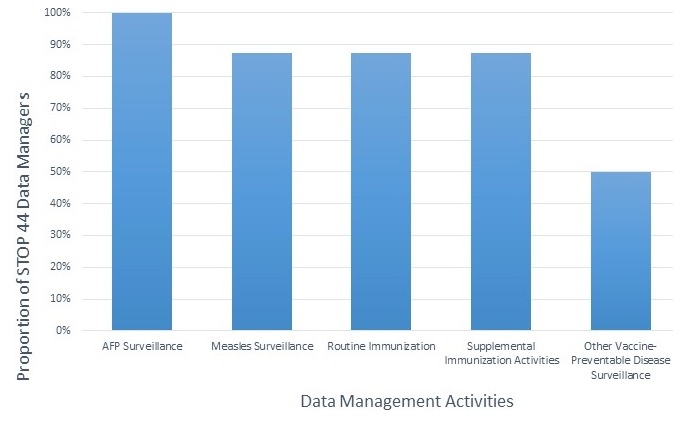
The proportion of STOP deployment number 44 data managers reporting to support data management activities by immunization data topic

STOP DMs are usually placed in the Expanded Program on Immunization (EPI) at the national level, and have played a critical role in developing system and human resource capacity to strengthen EPI immunization information and VPD surveillance systems. In 2014, a mixed-methods survey of STOP 44 DMs (N = 16) was conducted to better understand and describe STOP DM activities during field deployment. The results showed that STOP DMs engage in a broad range of activities to support immunization service delivery and VPD surveillance data management, with the most frequently reported activities being data analysis, data cleaning, and data quality control (Figure 1). While all STOP DMs supported acute flaccid paralysis (AFP) surveillance, the majority (14 of 16) also reported supporting other non-polio immunization activities (Figure 2).

### Evolution and future of STOP DM

Since the initial creation of the STOP DM deployment, significant changes have occurred, which have and are influencing the evolution of the program and future directions. In many countries, there has been a rapid increase in the use of integrated surveillance and health management information systems, while transitioning away from vertical immunization data systems. Similarly, information system software has transitioned to technologically more complex software platforms, such as DHIS2 [8], that necessitate development and maintenance by a workforce with expertise in information technology. As such, the role of national immunization data managers has commonly evolved to focus on end use of data, as opposed to collection and management of data from lower levels of the system. In addition, some countries began to rely on STOP DM assignees to ‘gap fill’ open data management positions within their country of assignment, as opposed to the goal of the STOP DM deployment to provide capacity development in immunization data management. The development of well-defined terms-of-reference and routine activity monitoring have becoming priorities of the STOP program in recent years, in order to ensure maximal sustainable impact of STOP DM deployments.

There has additionally been an increasing focus within the STOP DM program to encourage efforts towards addressing root causes of poor data quality and use issues, and a realization of the importance of the primary point of data collection, the health facility, on the overall quality of the data system. A primary focus of the “polio legacy” is “to ensure that the investments made to eradicate poliomyelitis contribute to further health goals” [1]. To address these needs, the training of STOP DM assignees has increased its focus on immunization and VPD surveillance data beyond polio, identification of root causes and implementing strategic and sustainable recommendations, and improving workforce capacity through mentorship and on-the-job support.

Finally, owing to the evolving nature of data needs in immunization programs, the STOP program has recently piloted an updated deployment, referred to as STOP Immunization and Surveillance Data Specialists (ISDS), with the specific focus of improving the quality and use of data at lower levels of the health system. In contrast to the traditional STOP DM deployment, STOP ISDS involves a team of data experts who deploy to a single country and are expected provide on-the-job mentorship at sub-national levels, including the district and health facility. The first ISDS deployment was launched in August 2016, in which 5 assignees were placed sub-nationally in Kenya.

### Conclusion

The STOP DM program has played a valuable role in ensuring the ability of countries in Africa to monitor performance of the GPEI. The program has adapted to changing needs of countries and will continue to evolve to meet future immunization and VPD surveillance data needs. Disclaimer: the findings and conclusions in this report are those of the authors and do not necessarily represent the views of the Centers for Disease Control and Prevention.

## Competing interests

The authors declare no competing interest.
